# Estabilidad postratamiento de la mordida abierta en pacientes con dentición permanente. revisión

**DOI:** 10.21142/2523-2754-1301-2025-232

**Published:** 2025-03-03

**Authors:** Pilar Vanessa Revilla-Márquez, Gissella Gutiérrez-Tapia

**Affiliations:** 1 División de Ortodoncia, Carrera de Estomatología, Universidad Científica del Sur. Lima, Perú. 100012394@cientifica.edu.pe ggutierrezt@cientifica.edu.pe Universidad Científica del Sur División de Ortodoncia Carrera de Estomatología Universidad Científica del Sur Lima Peru 100012394@cientifica.edu.pe ggutierrezt@cientifica.edu.pe

**Keywords:** estabilidad, mordida abierta, ortodoncia, tratamientos, dentición permanente, stability, open bite, orthodontic, treatments, permanent dentition

## Abstract

**Objetivo: Revisar,:**

en la literatura actual, la estabilidad a largo plazo de los diferentes tratamientos de ortodoncia para la corrección de la mordida abierta en pacientes con dentición permanente.

**Materiales y método.:**

Se realizó una búsqueda electrónica en cuatro bases de datos (PubMed, Elsevier, ScienceDirect, Google Scholar) y dos revistas especializadas (*AJODO, Angle Orthodontics*) hasta mayo de 2024. La calidad metodológica de los estudios fue analizada usando PRISMA checklist.

**Resultados:**

En esta revisión se incluyeron 22 estudios, que se analizaron de acuerdo con los diferentes tipos de tratamientos que brindan estabilidad a largo plazo para el cierre de la mordida abierta, como los tratamientos con y sin extracciones, el uso de minitornillos y miniplacas, y la cirugía ortognática.

**Conclusiones:**

Los pacientes tratados mediante extracciones presentaron mayor estabilidad postratamiento de la mordida abierta, según factores como apiñamiento, tipo de patrón vertical y control de hábitos orales. En los pacientes tratados con minitornillos o miniplacas se produce rotación antihoraria de la mandíbula después de la intrusión de los molares. La terapia miofuncional es necesaria para complementar la estabilidad de la mordida abierta, porque reeducará la postura de la lengua. Los pacientes de clase II sometidos a un tratamiento ortoquirúrgico tienen más tendencia a la recidiva por la musculatura y la poca adaptación del cóndilo.

## Abstract (en)

**Objective:**

The aim of this investigation is to review in the literature, the long-term stability of different orthodontic treatments to correct the open bite in patients with permanent dentition.

**Materials and Methods:**

An electronic search was made oin four databases (PubMed, Elsevier, Science Direct, Google Scholar) and two specialized journals (AJODO, Angle Orthodontics) until May 2024. The methodological quality of the studies was analyzed using the PRISMA checklist.

Results: In this literature review, 22 studies were included, the studies were analyzed according to the different kind of treatments which provide long term stability to correct open bite like treatments with and without extractions, use of miniscrews, miniplates and orthognathic surgery.

**Conclusions:**

Patients treated with extractions, presented a better stability of open bite, depending on elements like crowding, vertical pattern and control of oral habits. Patients treated with miniscrews or miniplates, the mandible suffer a counterclockwise rotation after molar intrusion. Myofunctional therapy is essential to complete the stability of anterior open bite because tongue rest posture will be reeducated. In skeletal class II patients treated with orthognathic surgery have a higher risk of relapse in the absence of adequate muscular and condylar adaptation.

## INTRODUCCIÓN

La mordida abierta es una de las maloclusiones que históricamente ha sido considerada un desafío desde su diagnóstico, tratamiento y postratamiento, el cual tiene una tendencia a la recidiva. La mordida abierta se define como una ausencia del traspase vertical entre los bordes incisales de los dientes anteriores de la maxila y la mandíbula, con rangos que van desde una mordida abierta leve a una severa (si se encuentra asociada con una displasia esqueletal). La prevalencia es del 1,5% al 11%, y varía entre las diferentes razas, con mayor presencia en la población negra (16,5%) y la caucásica (3,5%) ^1^^-^^6^. Su etiología es multifactorial, compleja y se desarrolla por la combinación de factores esqueletales, dentales, respiratorios (síndrome de cara larga), hábitos de succión, posición incorrecta de la lengua en reposo y deglución alterada; elementos que agravarán esta maloclusión y afectarán su estabilidad ^2^^-^^7^.

Sus características esqueletales y dentoalveolares incluyen un plano mandibular inclinado, ángulo goníaco obtuso, altura facial inferior aumentada, posición posterior y baja del hueso hioides, rotación antihoraria del plano palatino, planos oclusales divergentes, inclinación hacia mesial de los dientes posteriores con la posibilidad de una mordida cruzada posterior, sobreerupción de los molares superiores acompañada por la infraerupción y vestibularización de los incisivos ^3^^,^^8^^-^^12^.

Debido a las características y los factores etiológicos, el tratamiento de la mordida abierta varía entre los pacientes en crecimiento y los adultos ^4^, siendo el tratamiento durante la fase de dentición decidua o mixta el que presenta mejores resultados con menor tendencia a la recidiva ^13^^,^^14^. En pacientes adultos, el objetivo principal es asegurar la retención y la estabilidad a largo plazo durante el postratamiento ^3^^-5, 14-17, 18)^.

En el estudio de López *et al*. ^16^ evaluaron la estabilidad a largo plazo de la mordida abierta en pacientes con dentición permanente bajo el tratamiento de aparatología fija y arco extraoral, y determinaron que aproximadamente el 35% de los pacientes presentó 3 mm de recidiva. Otro estudio, el de Kim *et al*. ^17^, reportó el uso de arcos *multiloop* (MEAW), combinado con el uso de elásticos intermaxilares para conseguir intrusión de los molares y extrusión de los incisivos, a fin de cerrar la mordida abierta. Obtuvieron recidiva de solo un 6%, en un periodo postratamiento de 2 años.

Otro tipo es el tratamiento ortoquirúrgico, indicado para corregir la mordida abierta severa cuando hay un compromiso estético y es necesario una mayor impactación posterior de la maxila. Este tratamiento, a veces, no es aceptado por los pacientes por ser invasivo ^19^^,^^20^, con la introducción del anclaje esqueletal, minitornillos y miniplacas. También se consigue la intrusión de los dientes posteriores y la rotación de la mandíbula, con una estabilidad de la mordida abierta a largo plazo muy similar a la de la cirugía ortognática ^18^^,^^21^.

Se llega a un consenso entre toda la literatura que el tratamiento para la mordida abierta en pacientes con dentición permanente, sin o con poco crecimiento residual, es un desafío para el ortodoncista y puede tener poca estabilidad a largo plazo. Por tanto, el objetivo de este estudio será revisar y describir en la literatura actual la estabilidad a largo plazo de los diferentes tratamientos de ortodoncia para la corrección de la mordida abierta en pacientes con dentición permanente.

## MATERIALES Y MÉTODOS

Esta revisión de literatura fue registrada en la Universidad Científica del Sur con el número N.° 000490.

### Estrategia de búsqueda

La búsqueda del material bibliográfico fue ejecutada, hasta mayo de 2024, en 4 bases de datos: PubMed, Elsevier, ScienceDirect y Google Scholar, utilizando como palabras clave “estabilidad”, “mordida abierta”, “tratamientos”, “dentición permanente”. Adicionalmente, se buscó de forma manual en las revistas acreditadas *American Journal of Orthodontics and Dentofacial Orthopedics (AJODO)* y *Angle of Orthodontics*. Los criterios de búsqueda se describen en la Tabla 1. 

**Tabla 1:** Estrategia de búsqueda de descriptores de las diferentes bases de datos

Fuente	Fecha de búsqueda	Keywords	Resultados
PubMed	10/05/20244	(“stability” AND “open bite” AND “permanent dentition”)	14
Elsevier	10/05/2024	(“stability” AND “open bite” AND “permanent dentition”)	3
ScienceDirect	10/05/2024	(“stability” AND “open bite” AND “permanent dentition”)	2
Google Scholar	10/05/2024	(“stability” AND “open bite” AND “permanent dentition”)	14
*AJODO*	10/05/2024	(“stability” AND “open bite” AND “permanent dentition”)	11
*Angle Orthodontist*	10/05/2024	(“stability” AND “open bite” AND “permanent dentition”) Filters: Publication year from 2010 to 2023	3

### Proceso de calibración

El proceso de calibración fue realizado por el investigador principal (PRM) y el segundo autor (GGT). Se seleccionaron 22 artículos y se obtuvo un 100% de concordancia en la búsqueda de artículos y la extracción de los resultados según el interés.

### Recolección de datos

Los estudios recolectados se analizaron según su título y resumen. De no presentar resumen disponible, se evaluó el texto completo. El investigador principal revisó los artículos y su relevancia para esta investigación. En caso de dificultades para la selección, estas fueron resueltas por el segundo autor.

### Manejo de datos

El investigador sintetizó los datos de los estudios relacionados con el tema de investigación en la Tabla 2, donde se indican el autor, el año, los resultados y las conclusiones. Fueron 22 los artículos considerados para esta investigación.

**Tabla 2:** Extracción de la información

Autor y año	Diseño de estudio	Muestra	Resultados	Conclusiones
Kim *et al*. (2000)	Estudio de cohorte	Radiografías cefalométricas de 55 sujetos. En 2 grupos: el grupo en crecimiento (29) y el grupo en no crecimiento (26). Tratados con arcos Multiloop (MEAW), evaluados en periodo de 2 años postratamiento.	El overbite aumentó en ambos grupos en 4 mm y, en la fase postratamiento de 2 años, se mantuvo estable, sin cambios significativos.	La corrección de la mordida abierta se dio principalmente por la extrusión de los incisivos con el uso de elásticos intermaxilares.
Janson *et al*. (2003)	Estudio retrospectivo	Radiografias cefalométricas de 21 sujetos Primer grupo con mordida abierta y el otro con normoclusión. Evaluados en 5 años postratamiento.	Correlación entre la disminución del overbite después del tratamiento y el incremento de la altura dentoalveolar de los molares inferiores en el periodo posretención. A pesar de eso, el 61,9% de la muestra mantuvo un overbite estable a largo plazo.	La disminución del overbite en el periodo posretención se produce por el poco crecimiento del hueso en el sector anterior y el mayor crecimiento de la altura dentoalveolar de los dientes posteriores.
De Freitas *et al*. (2004)	Estudio cohorte	Radiografías cefalométricas. El grupo experimental de 31 pacientes tratados con extracciones y el grupo control de 36 sujetos. Se evaluó de T1 a T3 en un periodo de 8,35 años.	La disminución del overbite fue estadistícamente no significativa en el período de T3. Estabilidad del 74,2% en el período de 8 años posretención (T3) en el grupo experimental.	El desarrollo vertical de los incisivos superiores e inferiores, y el poco desarrollo vertical de las molares inferiores contribuyeron a que el overbite se mantenga estable en el grupo experimental en la evaluación en T3.
Janson *et al*. (2006)	Estudio de cohorte	Radiografías cefalométricas, grupo 1 de 21 sujetos tratados sin extracciones y grupo 2 de 31 sujetos con extracciones. El periodo de posretención en el grupo 1 fue de 5,22 años y en el grupo 2, de 8,35 años.	En el periodo de posretención, el grupo 1 presentó mayor desarrollo vertical de los incisivos superiores e inferiores, y una mayor disminución del overbite que el grupo 2. El overbite disminuyó (-0,06 mm) en el grupo 2 en T3.	El overbite se mantiene más estable en el periodo de posretención en el grupo que se realizó extracciones.
Remmers *et al*. (2008)	Estudio retrospectivo	Radiografías cefalométricas de 52 sujetos, 17 tuvieron extracciones de premolares. El seguimiento postratamiento fue un promedio de 8,3 años.	37 pacientes presentaron un overbite positivo al final del tratamiento. De estos, 10 presentaron una recidiva en el postratamiento.	La estabilidad a largo plazo de la mordida abierta no tiene factores predecibles, y esta dependerá de cambios funcionales y el tipo de crecimiento después del tratamiento.
Espeland *et al*. (2008)	Estudio retrospectivo	Evaluación cefalométrica de la estabilidad de la osteotomía Lefort 1 con fijación rígida de 40 sujetos con un período de seguimiento de 3 años. El overbite promedio antes de la cirugia fue de 2,6 mm.	En el postratamiento, 35 presentaron un overbite positivo y los 5 restantes tuvieron una recidiva entre 0,2 y 0,9 mm. Los que tuvieron una impactación anterior de la maxila mayor a 2 mm, presentaron una recidiva del 62% .	La mayor recidiva se presentó en los primeros 6 meses posquirúrgico. Un overbite estable en el periodo de seguimiento despues de 3 años y los cambios verticales del maxilar se compensaron con la ortodoncia posquirúrgica.
Smithpeter *et al*. (2010)	Estudio de cohorte	76 sujetos, 27 (grupo experimental) recibieron terapia miofuncional con tratamiento o retratamiento de ortodoncia. 49 (grupo control) tuvieron solo tratamiento de ortodoncia.	En el grupo experimental, 17 no presentaron recidiva, 9 presentaron un overbite de -1 mm y 1 recidiva de -4 mm. 74% del grupo control presentó hábitos orales de higiene, la recidiva promedio fue de -3,38 mm.	La recidiva en el grupo experimental fue menor de 0-4 mm, a diferencia del grupo control, que varió entre 1 y 7 mm. Se corrigió la postura de la lengua en reposo y deglución, la pronunciación de algunas fonemas y hábitos.
Baek *et al*. (2010)	Estudio prospectivo	El estudio evaluó la impactación posterior maxilar de 9 sujetos tratados con anclaje esqueletal, en un periodo postratamiento de 1año (T3) y 3 años (T4).	Al final del tratamiento, el overbite aumentó de -3,91 a 1,65 mm. En el periodo de T4, el overbite disminuyó 1,20 mm y la distancia del plano palatino a la molar superior aumentó un promedio de 1,20 mm.	La corrección se presenta por una rotación horaria de la mandíbula. Existe una correlación positiva entre la cantidad de corrección durante el tratamiento y la recidiva postratamiento.
Janson *et al*. (2010)	Estudio retrospectivo	Radiografías cefalométricas después de 3,4 años, de 17 sujetos, que presentaron recidiva de la mordida abierta y fueron tratados nuevamente con ajustes oclusales.	En el postratamiento se observó una pequeña recidiva en la altura facial anteroinferior y el overbite. Un 66,7% de la muestra presentó una estabilidad del overbite a largo plazo.	La recidiva de la mordida abierta se presentó por el incremento de la altura en las molares superiores. Se puede presentar una mayor estabilidad después de realizar el ajuste oclusal en pacientes mayores de 21 años,
Zuroff *et al*. (2010)	Estudio de cohorte	Radiografías cefalométricas de 64 pacientes, evaluados postratamiento de 9,5 años (T3). G1(n = 24) con contacto incisal,G2 (n = 25) con sobrepase horizontal sin contacto incisal y G3 (n = 15) con mordida abierta.	En T3, G3 presentó un overjet mayor que G1, pero el overbite se mantuvó estable en el grupo G1. El 20% de los sujetos de G3 presentaron menor recidiva vertical, pero todos presentaron un sobrepase horizontal.	Toda la muestra presentó un overbite positivo al final del tratamiento, pero, en la evaluación a largo plazo, el 60% del grupo de mordida abierta presentó falta de contacto incisal.
Silva *et al*. (2012)	Estudio de cohorte	Evaluación de modelos de yeso de 33 pacientes sometidos a una osteotomía Le Fort 1 en períodos de 6, 12, 18 y 30 meses poscirugía.	En la evaluación a 30 meses, hubo una recidiva de 2-3,5 mm en el 67% de pacientes donde el maxilar fue movido más de 7 mm. Un 11% de pacientes tuvieron recidiva cuando el maxilar se movió verticalmente 7 mm. Sin recidiva cuando el maxilar se movió menos de 4 mm.	Después de la cirugía, se necesita que haya un trabajo junto a la ortodoncia para compensar la recidiva con movimientos dentoalveolares.
Tettinen *et al*. (2012)	Estudio retrospectivo	Evaluación cefalométrica de 24 pacientes en un periodo posquirúrgico entre 2-3,5 años (T3). 12 con reposicionamiento superior de la maxila (OB inicial -2,6 mm) y 12 (OB inicial -2,2 mm) con una impactación de la maxila + ostetomía mandibular.	En (T3) el overbite fue de 1,85 mm en el primer grupo y de 0,73 mm en el segundo grupo. Un overbite negativo en 3 sujetos que recibieron cirugía bimaxilar.	Menor estabilidad a largo plazo cuando la cirugía fue bimaxilar, por el crecimiento vertical de la maxila y la rotación horaria de la mandíbula. Es ligeramente más estable cuando solo se trata la maxila.
Geron *et al*. (2013)	Estudio retrospectivo	Evaluación clínica y cefalométrica de 39 pacientes tratados con brackets linguales en un período promedio posretención de 4,01 años (T3).	De los 39 sujetos, el 12,5% presentó recidiva en el overbite. Entre el periodo de finalizado el tratamiento y el de posretención, se presentó un incremento significativo del OB de 2 a 2,5 mm.	El tratamiento con brackets linguales permitió el control de la posición de la lengua, lo cual permitió que el overbite se mantuviera estable en el periodo de posretención.
Scheffler *et al*. (2014)	Estudio retrospectivo	33 pacientes tratados con una ferúla oclusal maxilar y minitornillos ubicados en el área infracigomáticas. 27 sujetos evaluados después de 1 año y 25 evaluados después de 2 años.	En el periodo posretención de 1 año, el overbite disminuyó 0,3 mm y las molares superiores extruyeron 0,5 mm. Después de 2 años, en el 16% de la muestra, las molares superiores se extruyeron 2-4 mm.	La intrusión de las molares superiores, de 5-6 mm, apoyado en minitornillos, es de gran ayuda para la corrección de la mordida abierta.
Salehi *et al*. (2015)	Estudio de cohorte	Evaluación cefalométrica de 37 pacientes tratados con extracciones y sin extracciones. El overbite fue entre 0 y -3 mm. La evaluación posretención fue entre 3 y 6 años (T3).	En el periodo de T3, el overbite promedio fue de -0,46 mm y 6 pacientes presentaron mordida abierta.	No hay diferencia significativa en los grupos de extracciones y sin extracciones. Los retenedores fijos no aseguran la estabilidad a largo plazo de la mordida abierta.
Marzouk *et al*. (2016)	Estudio de cohorte	Evaluación cefalométrica de 26 pacientes tratados con miniplacas en la región cigomática. El overbite inicial (T1) varió entre -3 a -8 mm, la evaluación postratamiento fue de 4 años (T4).	La intrusión molar fue de 3,04 mm y un incremento promedio del overbite de 6,49 mm. Los índices de recidiva en T4 de la intrusión molar fueron del 13,37% y del overbite, del 11,18%.	Se presentó una correlacion positiva en la intrusion molar y el overbite postratamiento con la cantidad pretratamiento. La estabilidad se produjo por la cantidad de intrusión molar y la extrusión de incisivos superiores.
Vela Hernández *et al*. (2017)	Estudio retrospectivo	Evaluación cefalométrica de 31 pacientes tratados con bloques de resina en las cúspides palatinas de molares superiores. La evaluación posretención fue un tiempo promedio de 32,9 meses (T3).	El overbite se incrementó postratamiento en 3,98 mm y tuvo una recidiva en T3 de 0,56 mm. Se produjó una intrusión molar de 1,5 mm y una recidiva en T3 de 0,10 mm.	Los topes de resina ayudan en la corrección de la mordida abierta, por la intrusión de las molares superiores, y producen una rotación antihoraria de la mandíbula
Malara *et al*. (2021)	Revision sistemática	Estudio de 10 artículos: 5 tratados con minitornillos, 5 ortoquirúrgicos	La recidiva despues de 3 años postratamiento fue por la rotacion horaria mandibular. Cuando la cirugía fue bimaxilar, hubo disminución del overbite, a diferencia de la cirugía Lefort 1, donde el overbite aumentó 0,62 mm después de 3,5 años.	Se presentó más estabilidad cuando la cirugía fue solamente maxilar.
Elhajoubi *et al*. (2021)	Revision sistemática	Estudio de 5 artículos de tratamientos ortoquirúrgicos	La mayor recidiva despues de 3,5 años se presentó cuando la cirugía fue bimaxilar, en comparacion cuando la intervención solo fue a nivel del maxilar.	Mayor estabilidad a largo plazo en cirugía maxilar por la impactación posterior y la reposición anterosuperior. Los pacientes de clase II presentan mayor recidiva.
Gu *et al*. (2022)	Estudio de cohorte	112 pacientes tratados con alienadores, aparatología fija, minitornillos y quirúrgico. Evaluados con fotos intraorales en un periodo promedio postratamiento de 1,21 años.	Los tratados con extracciones y poca inclinación del incisivo inferior mostraron mayor estabilidad.	No hubo diferencias significativas en la estabilidad entre los grupos. Los retenedores termoplastificados presumen tener un efecto intrusivo al cubrir las caras oclusales.
Theodoridou *et al*. (2023)	Revision de literatura	Estudio de 5 artículos	El ángulo nasolabial disminuyó por la retroinclinación de los incisivos y el uso de minitornillos produjo la intrusión en molares.	En los tratamientos no quirúrgicos, los cambios se dan principalmente a nivel dentoalveolar, como la intrusión de molares, extrusión y retroinclinación de incisivos superiores e inferiores.
Hammad *et al*. (2023)	Estudio retrospectivo	23 pacientes tratados con arcos de extrusión de acero y evaluados con radiografías cefalométricas 12 meses después de finalizado el tratamiento.	El overbite fue estable en 1,40 mm promedio después de 1 año de tratamiento.	Este tipo de arcos de extrusión son ideales para pacientes con poca exposición de incisivos en reposo y sonrisa.

**Figura 1:**
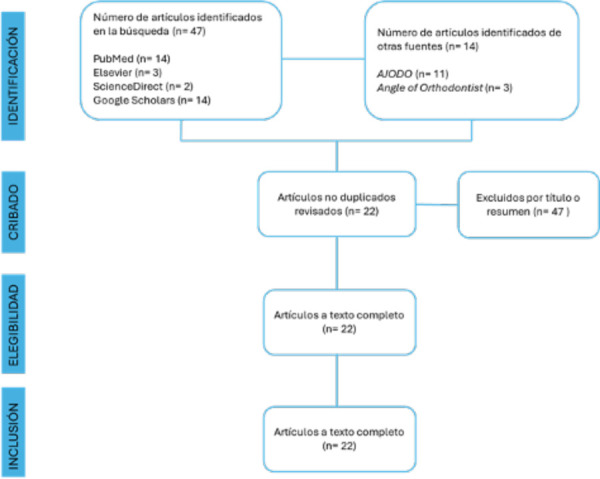
Diagrama de flujo PRISMA de la revisión de la literatura

## RESULTADOS

### Terapia miofuncional

Esta terapia consta de una serie de ejercicios que reeducan los músculos orofaciales en la deglución, el habla y el reposo ^5^^,^^22^. Varios autores declaran que esta terapia es útil como complemento al tratamiento de ortodoncia; sin embargo, los beneficios que presenta son cuestionables para muchos ^22^.

Las razones negativas que mencionan para que esta terapia no se pueda incluir en la práctica ortodóntica son las siguientes ^22^:

• Espacio limitado en el consultorio para realizar las terapias.

• Pocos terapistas calificados y entrenamiento inadecuado por parte del ortodoncista.

• Dificultad para el paciente al realizar los ejercicios y poco tiempo para realizarlos.

• Poca evidencia científica que respaldan estas terapias.

• No todos los terapistas tienen la suficiente experiencia para lograr resultados exitosos, lo que lleva a la duda en el pronóstico del tratamiento.

Smithpeter *et al*. ^22^ realizaron un estudio con dos grupos de individuos que presentaban recidiva de mordida abierta después de haberse realizado un tratamiento de ortodoncia. El grupo experimental recibió tratamiento de ortodoncia y terapia miofuncional. En los resultados, la recidiva fue de 0 a 4 mm para este grupo, a diferencia del grupo control, que solo recibió tratamiento de ortodoncia y tuvo una recidiva de 1 a 7 mm.

Los estudios de Kim *et al*. ^17^ y Janson *et al*. ^23^, sobre la corrección de la mordida abierta sin extracciones, indican que, para asegurar la estabilidad de la mordida abierta, es imprescindible la corrección de la postura de la lengua en reposo y terapia de lenguaje antes, durante y después del tratamiento. Asimismo, De Freitas *et al*. ^14^, Janson *et al*. ^24^ y Gu *et al*. ^25^ indican que, para brindar la estabilidad en pacientes tratados con extracciones, no solo se necesita el uso de retenedores activos (*bite blocks*) o retenedores fabricados en el vacuum al finalizar el tratamiento, sino complementarlos con terapia de lenguaje.

### Tratamiento sin extracciones

En el 2000, Kim *et al*. ^17^, Janson *et al*. ^23^ y Theodoridou *et al*. ^26^ encontraron que la corrección de la mordida abierta se produjo por una extrusión y verticalización de los incisivos superiores e inferiores, con una mejora en los tejidos blandos, específicamente en el ángulo nasolabial, el cual tuvo una disminución favorable. Esta extrusión se presentó en la fase de nivelación, por el uso de elásticos intermaxilares que ayudan al cierre de la mordida y de rejillas linguales para corregir la succión digital y la deglución adaptada, dispositivos utilizados en el estudio de Theodoridou. La diferencia que presentaron los dos primeros estudios mencionados fueron la cantidad de años en la fase de postratamiento para evaluar la estabilidad, que fue de 5 años en el estudio de Janson y de dos años en el de Kim. En este último, la evaluación postratamiento se realizó en 27 sujetos, 28 menos que su muestra inicial.

Para la estabilidad en la corrección de la mordida abierta, también se necesita de la intrusión de las molares ^17^, el estudio de Vela Hernández *et al*. ^27^ utilizó topes oclusales de resinas de 2 a 3 mm en las cúspides funcionales de los molares superiores, los cuales se intruyeron 1,5 mm durante el tratamiento, con una recidiva de solo 0,10 mm en el periodo de retención que fue entre 12 y 82 meses. En cambio, en el estudio de Zuroff *et al*. ^28^ utilizaron aparatología Edgewise y la muestra se dividió en tres grupos: el grupo de contacto incisal, el grupo de sobrepase y el grupo de mordida abierta. Este último tuvo una recidiva no estadísticamente significativa de 0,40 mm en el 21% de los pacientes durante el periodo de postratamiento, lo que se atribuyó a la erupción de los molares sin la extrusión de los incisivos.

Según los estudios de Janson ^23^, Kim ^16^ y Vela Hernández ^27^, otro factor que ayuda al cierre de la mordida es la corrección de los planos oclusales, en especial del plano inferior (mandibular), porque está rota en sentido antihorario, lo que hace más estable a largo plazo el cierre de la mordida abierta.

Otro estudio sin extracciones para la corrección de la mordida abierta fue el de Janson *et al*. ^29^, en el cual el tratamiento se realizó con ajustes oclusales según el método de Okeson a pacientes que presentaron recidiva de la mordida abierta (OB -1,11 mm) que tuvieron tratamiento previo de ortodoncia. La estabilidad del overbite tres años después de los ajustes oclusales fue del 66,7% en el total de la muestra, y la recidiva del overbite fue de 0,76 mm. En los pacientes donde se realizaron los ajustes oclusales, se concluyó que el factor principal que contribuye a la recidiva es la extrusión del sector posterior, en un intento de restablecer el balance muscular fisiológico. 

En el reciente estudio de Hammad *et al*. ^30^, en pacientes que presentaban un overbite inicial promedio (T1) de -3,8 mm, el tratamiento consistió en la colocación de arcos de extrusión de acero de .016 x .022”, los cuales fueron ligados al sector anterior para producir un solo sistema de fuerza. La corrección se produjo principalmente por una extrusión de los incisivos superiores e inferiores, donde el overbite se mantuvo estable (T2) +1,40 mm a los 17 meses postratamiento. Los autores recalcaron que estos arcos de extrusión son ideales para pacientes que presentan poca exposición de incisivos en reposo y en sonrisa.

### Tratamiento con extracciones

Los estudios en los cuales se realizaron extracciones de premolares para la corrección de la mordida abierta presentaban apiñamiento o un patrón vertical más acentuado. 

Tal es el caso del estudio de De Freitas *et al*. ^14^, donde se observó una estabilidad en el 74% de los pacientes después de un periodo de ocho años, y el overbite presentaba un promedio de 1,02 mm en el periodo posretención, lo que coincide con los hallazgos de Kim *et al*. ^17^ y con el estudio de Janson *et al*. ^24^ donde la estabilidad del overbite, después de un periodo postratamiento de 5 años, fue de 1,03 mm en el 74,2% de los pacientes.

La corrección de la mordida abierta se debió principalmente a la retrusión, extrusión y verticalización de los incisivos superiores, debido al cierre de espacios y el uso de los elásticos intermaxilares, lo que coincide con los resultados en los estudios de Janson *et al*. ^24^ y Kim *et al*. ^17^. Sin embargo, la poca extrusión de los incisivos inferiores por una mayor retracción y lingualización generaron una mayor estabilidad postratamiento y un mejor cierre de la mordida abierta.

Se presentaron dos estudios de Remmers *et al*. ^31^ y de Geron *et al*. ^32^, que analizaron grupos con extracciones y sin extracciones. En el primero no se encontró una asociación del cierre de la mordida abierta con el tratamiento mediante extracciones, ni al final del tratamiento ni en la evaluación después de 8 años, en comparación con el estudio de Geron, donde, en el grupo con extracciones, el overbite aumentó entre el final del tratamiento y la evaluación postratamiento luego de 11 años.

No obstante, el tratamiento con extracciones no asegura una estabilidad a largo plazo porque dependerá de factores como el apiñamiento, el patrón vertical, los hábitos orales y el tipo de contención que se utilizará una vez finalizado el tratamiento, como en el estudio de Salehi *et al*. ^33^, donde se presentó recidiva a pesar del uso de retenedores ^23^^, 31)^.

Al tratarse de un tratamiento con extracciones, se puede presentar recidiva y no solo será necesario el uso de retenedores al finalizar el tratamiento, sino también de terapia miofuncional, como indican los estudios de Smithpeter *et al*. ^22^ y Janson *et al*. ^23^, los cuales afirman que, para la corrección y estabilidad de la mordida abierta, es imprescindible la corrección de la postura de la lengua y la terapia de lenguaje antes, durante y después del tratamiento ^14^^,^^17^^,^^27^.

### Tratamiento con minitornillos y miniplacas

El uso del anclaje esqueletal, como minitornillos y miniplacas, ha sido introducido hace poco tiempo para realizar movimientos ortodóncicos sin la necesidad de la cooperación del paciente. El primer reporte del uso de anclaje esqueletal fue en 1983, por Creekmore y Euklund ^34^, quienes colocaron un tornillo encima de la espina nasal anterior para intruir los incisivos superiores. Umemmori y Sugawara ^35^, en 1999, fueron los primeros en utilizar el anclaje esqueletal para la intrusión de las molares superiores. Ellos introdujeron el sistema de anclaje esqueletal (SAS), que consistía en el uso de miniplacas quirúrgicas localizadas en la parte posterior de la mandíbula para intruir los molares y corregir la mordida abierta. 

En el estudio de Baek *et al*. ^21^ utilizaron, en un primer grupo, minitornillos solo por vestibular entre primer y segundo premolar, y entre primer y segundo molar acompañados por una barra transpalatina para prevenir la inclinación de la corona hacia vestibular al momento de la intrusión. En el segundo grupo, por vestibular y palatino entre primer y segundo molar. En los resultados, la distancia del molar al plano palatino disminuyó en 2,39 mm, resultado similar al de Scheffler *et al*. ^18^ donde la intrusión fue de 2,3 mm en el 60% de los pacientes, a los cuales les colocaron una férula de intrusión (AOB) apoyada en los premolares y molares, y los minitornillos entre el primer molar y el segundo premolar. Después del periodo de 3 años postratamiento, en el estudio de Baek, se presentó 0,45 mm de erupción del molar, y en los hallazgos de Scheffler de una muestra de 30 pacientes solo cuatro presentaron recidiva en la extrusión del molar.

En los reportes de Baek *et al*. ^21^ y Scheffler *et al*. ^18^, el overbite solo recidivó un 17% y un 22% respectivamente, de la muestra total de pacientes. La corrección del overbite se produjo por la rotación antihoraria de la mandíbula después de la intrusión ^17^^,^^24^^,^^27^^,^^36^.

Otro tipo de anclaje esqueletal utilizado son las miniplacas ^37^^,^^38^, dispositivos con lo que también se logran la intrusión de los molares superiores ^18^^,^^21^. En el estudio de Marzouk *et al*. ^38^, las miniplacas se colocaron en el arco cigomático y la intrusión fue generada por un resorte de niti que provocó una fuerza de 450 gramos, de modo que se produjo una intrusión de 3,04 mm de los molares superiores, mayor en comparación con los estudios con minitornillos.

Esta mayor intrusión de las molares empleando las miniplacas genera que la recidiva del overbite sea menor solo en un 11,18% en comparación con el uso de minitornillos ^38^.

Con el uso de las miniplacas, al igual que con los minitornillos, se consigue una rotación antihoraria de la mandíbula, siendo uno de los factores que permiten el cierre de la mordida ^18^^,^^21^^,^^38^.

La extrusión de los incisivos superiores se produce después de la intrusión de las molares, por lo que es un elemento importante para mantener la estabilidad en el periodo postratamiento ^18^^,^^21^. Sin embargo, en el estudio de Marzouk *et al*. ^38^ atribuyeron la estabilidad, a la correlación que se presenta entre la cantidad de intrusión de los molares superiores y la corrección del overbite.

Como se reportó en los estudios anteriores, la terapia miofuncional y la contención activa con férulas o retenedores circunferenciales con rejilla palatinas, después del tratamiento con dispositivos de anclaje temporal (TAD), juegan un rol muy importante para la estabilidad de la mordida abierta ^18^^,^^21^^,^^38^, especialmente después de realizar la intrusión de los molares superiores, para también evitar la extrusión de los molares inferiores.

### Tratamiento con cirugía ortognática

Uno de los factores etiológicos más comunes en la mordida abierta esqueletal es un mayor desarrollo vertical de la parte posterior de la maxila. Para lograr una verdadera intrusión de los dientes posteriores, se necesita la combinación de tratamiento ortoquirúrgico. Uno de los tratamientos quirúrgicos más utilizados es el LeFort 1, cuyo propósito principal es la impactación o reposicionamiento de la zona posterior de la maxila. Esto permite la rotación antihoraria de la mandíbula, lo que disminuye la altura facial anterior e incrementa el overbite ^39^^-^^42^. Lo señalado coincide con los resultados de Espeland *et al*. ^20^, que muestran una tendencia de aumento gradual del overbite después de realizada la cirugía. El estudio de Silva *et al*. ^39^, en el que 33 pacientes fueron tratados mediante Lefort 1 segmentaria, tras la evaluación a 2,5 años del tratamiento ortoquirúrgico, se observó una recidiva de la mordida abierta de 2-3,5 mm en el 67% de los pacientes, y la maxila fue movida más de 7,5 mm en sentido horizontal o vertical. La recidiva se presentó en el 11% de los pacientes y la maxila se movió más de 7 mm en sentido vertical. 

En el estudio de Teittinen *et al*. ^40^, la muestra se dividió en dos grupos: el primero por reposicionamiento superior de la maxila posterior mediante Lefort 1 (grupo maxilar) y el segundo mediante Lefort 1 más osteotomía sagital bilateral de la rama mandibular (grupo bimaxilar). En ambos grupos, en el periodo postratamiento de 3 años, la maxila recidivó en sentido vertical (rotación antihoraria), pero el cambio fue estadísticamente significativo en el grupo maxilar por la disminución del ángulo SN-Max de 9,59° a 7,45°. 

Otro estudio donde también se realizó la cirugía fue de Lefort 1 es el de Malara *et al*. ^43^, donde, en el grupo que presento cirugía solo con Lefort 1, el overbite aumentó en 0,62 mm después de 3,5 años postratamiento, a diferencia del grupo que presento cirugía bimaxilar, donde el overbite disminuyó a 0,25 mm con el mismo tiempo postratamiento.

El procedimiento quirúrgico con menor estabilidad es la cirugía mandibular, también llamada osteotomía sagital bilateral de la rama mandibular (BSSO), cuyo propósito es producir una rotación antihoraria de la mandíbula. Así lo reportó el estudio de Ajalmar *et al*. ^41^, ya que, en el periodo postratamiento de dos años, se presentó una recidiva del overbite en promedio de -3,13 mm en pacientes con relación esquelética clase II (por la rotación horaria de la mandíbula), a diferencia de los pacientes tratados con Lefort 1, para quienes la recidiva del overbite fue de 0,72 mm. 

Los cambios en el componente dentoalveolar después de la cirugía, como la proinclinación de los incisivos inferiores y la verticalización de los incisivos superiores, permitieron que el overbite y la corrección de la mordida abierta se mantuvieran estables durante el periodo postratamiento ^42^.

Un factor etiológico importante como la relación esquelética tuvo influencia con la recidiva de la mordida abierta, de acuerdo al estudio de Teittinen *et al*. ^40^ y Elhajoubi *et al*. ^44^. Los pacientes de clase II con síndrome de cara larga presentaron mayor recidiva, porque precisaron un mayor avance mandibular y adaptación muscular después de la cirugía, con lo que se convierte en un gran factor de riesgo para la estabilidad a largo plazo, a diferencia de los pacientes de clase III del estudio de Ajalmar *et al*. ^41^, en el cual el overbite fue más estable en el 87,50% de la muestra.

## DISCUSIÓN

El propósito de este estudio es brindar información actualizada acerca de la estabilidad postratamiento de la mordida abierta tratada con mioterapia, con y sin extracciones, anclaje esqueletal y cirugía ortognática, para lo que se realizó una revisión de la literatura. Sin embargo, la cantidad de pacientes tratados y observados a largo plazo es limitada.

Los artículos analizados en esta revisión reportaron que hay una relación directa entre la terapia miofuncional y la estabilidad de la mordida abierta a largo plazo, por lo que esta es una herramienta importante si forma parte del tratamiento, ya que ayuda a la correcta postura de la lengua en reposo antes, durante y después del tratamiento ^17^^,^^23^.

En cuanto a los tratamientos con y sin extracciones -aparte de la extrusión de los incisivos en las primeras fases del tratamiento-, la intrusión de las molares superiores e inferiores, y la corrección del plano oclusal en sentido antihorario serán los factores que brindarán la estabilidad a largo plazo en la corrección de la mordida abierta ^17^^,^^23^^,^^27^. También se reportó que los tratamientos con extracciones no aseguran la estabilidad, ya que esta dependerá del grado de apiñamiento, el patrón vertical, los hábitos orales y el tipo de retenedor utilizado ^23^^,^^31^^,^^33^.

Según la literatura actual, los minitornillos y las miniplacas son los más utilizados por la poca cooperación del paciente y el apoyo en el hueso inter o extrarradicular. Los minitornillos fueron utilizados para intruir las molares superiores, y se colocaron en posición vestibular o palatina. La recidiva que presentaron fue de 0,45 mm en la erupción de la molar, después de la evaluación de 3 años. Al producirse esta intrusión, se corrige el overbite por la rotación antihoraria de la mandíbula y este recidivó en promedio entre un 17 y un 22% ^18^^,^^21^^,^^36^.

Cuando la mordida abierta es de tipo esqueletal con un patrón vertical importante, se realiza la combinación de cirugía ortognática y el tratamiento de ortodoncia. Como en los tratamientos anteriores se necesita una impactación de la zona posterior de la maxila, el tipo de cirugía elegida es el Lefort 1 ^20^^,^^39^^-^^41^. Cuando se necesita impactar la maxila más de 7 mm, la estabilidad disminuye un 11%. Otro factor importante en la estabilidad es la adaptación muscular, y son los pacientes clase II esqueletal con aumento en la altura facial los que precisan un mayor avance de la mandíbula y, por lo tanto, una mayor adaptación después de la cirugía ^40^^,^^41^^,^^44^.

Los artículos originales y las revisiones sistemáticas han encontrado limitaciones al momento de elaborar las conclusiones por la muestra limitada al evaluar la estabilidad en un periodo mayor de 3 años, ya que es cuando la población inicial de la investigación disminuye al no poder realizar el seguimiento del estudio.

### Limitaciones:

Como limitaciones de esta revisión tenemos el escaso número de artículos en los cuales se evalúa la estabilidad a largo **plazo en una muestra grande, lo que dificulta obtener resultados significativos.**

## CONCLUSIONES

1. Los tratamientos con extracciones presentan mayor estabilidad a largo plazo en la corrección de la mordida abierta, pero dependen de factores como el grado de apiñamiento, el tipo de patrón vertical y el control de los hábitos orales del paciente.
2. Finalizado el tratamiento, es necesaria la terapia miofuncional para la reeducación de la postura de la lengua, y un protocolo de contención que evite la extrusión de los molares, con el fin de asegurar la estabilidad a largo plazo.
3. El uso de minitornillos o miniplacas produce la intrusión de los molares superiores e inferiores, y la extrusión de los incisivos, lo que genera la rotación antihoraria de la mandíbula. Estos elementos ocasionan el cierre de la mordida abierta.
4. La cirugía Lefort 1 es uno de los tratamientos quirúrgicos más estables, ya que impacta en la zona posterior de la maxila; sin embargo, la estabilidad dependerá de la adaptación muscular.
5. Los pacientes con una relación esquelética de clase II que son sometidos a tratamiento ortoquirúrgico presentan mayor recidiva por el tipo de musculatura y la poca adaptación del cóndilo a la nueva postura de la mandíbula. Dicho tratamiento deberá ser complementado con un fisioterapeuta calificado, un elemento importante para favorecer la adaptación muscular.

## Financiamiento:

Autofinanciado
